# ginmappeR: an unified approach for integrating gene and protein identifiers across biological sequence databases

**DOI:** 10.1093/bioadv/vbae129

**Published:** 2024-08-29

**Authors:** Fernando Sola, Daniel Ayala, Marina Pulido, Rafael Ayala, Lorena López-Cerero, Inma Hernández, David Ruiz

**Affiliations:** SCORE Lab, DEAL, University of Seville, ETSII, 41012 Seville, Spain; SCORE Lab, DEAL, University of Seville, ETSII, 41012 Seville, Spain; Department of Microbiology, University of Seville, 41009 Seville, Spain; Institute of Biomedicine of Seville, Virgen Macarena University Hospital, CSIC, University of Seville, 41013 Seville, Spain; Centro de Investigación Biomédica en Red en Enfermedades Infecciosas (CIBERINFEC), 28029 Madrid, Spain; Molecular Cryo-Electron Microscopy Unit, Okinawa Institute of Science and Technology Graduate University, Okinawa 904-0411, Japan; Department of Microbiology, University of Seville, 41009 Seville, Spain; Institute of Biomedicine of Seville, Virgen Macarena University Hospital, CSIC, University of Seville, 41013 Seville, Spain; Centro de Investigación Biomédica en Red en Enfermedades Infecciosas (CIBERINFEC), 28029 Madrid, Spain; SCORE Lab, DEAL, University of Seville, ETSII, 41012 Seville, Spain; SCORE Lab, DEAL, University of Seville, ETSII, 41012 Seville, Spain

## Abstract

**Summary:**

The proliferation of biological sequence data, due to developments in molecular biology techniques, has led to the creation of numerous open access databases on gene and protein sequencing. However, the lack of direct equivalence between identifiers across these databases difficults data integration. To address this challenge, we introduce *ginmappeR*, an integrated R package facilitating the translation of gene and protein identifiers between databases. By providing a unified interface, *ginmappeR* streamlines the integration of diverse data sources into biological workflows, so it enhances efficiency and user experience.

**Availability and implementation:**

from Bioconductor: https://bioconductor.org/packages/ginmappeR

## 1 Introduction

Developments in large-scale molecular biology techniques such as next-generation sequencing (Goodwin *et al.* 2016, Satam *et al.* 2023), microarrays, and other omics techniques (Zhang *et al.* 2019) have led to an explosion in the amount of available biological sequences. This has prompted the creation of multiple databases where researchers deposit the sequences identified through a variety of experimental methods. These include, among others, sequences of both genes and proteins.

A large amount of modern bioinformatics workflows (Jacobsen *et al.* 2020, Reiter *et al.* 2021) typically involve integrating different sources of information that refer to these entities by unique identifiers within each source. That is why, nowadays, biological sequence databases offer public programmatic interfaces (APIs) to access their data, like NCBI (https://www.ncbi.nlm.nih.gov/), UniProt (https://www.uniprot.org/), or KEGG (https://www.kegg.jp/). Consequently, community developed R packages to consume these services are available, such as rentrez (Winter 2017), UniProt.ws (Carlson and Ramos 2023), and KEGGREST (Tenenbaum 2023). Other databases, like CARD (https://card.mcmaster.ca/), offer their data as a downloadable file.

However, there is no direct equivalence between the multiple sequence identifiers included in the different databases, which can lead to confusion for their users when searching for information on a particular gene or protein, as well as a considerable investment of time in the search for equivalences between databases. The heterogeneity and low coupling of these tools (Wieder *et al.* 2021) cause the user’s attention to be diverted from the main task in order to learn minimally how to use each of them. In addition, as free public services, they are prone to a variety of errors, such as random service denial errors or empty responses, that should be specifically controlled by the user.

These reasons motivated us to conceive *ginmappeR*, an integral R package that translates gene or protein identifiers between the mentioned databases, making it easier for users to work with multiple data sources in a unified and complete way. With additional features like UniProt similar genes and NCBI identical proteins retrieval, that enrich the translation process and are highly-demanded for studying gene and protein similarities, their roles across species and their evolutionary relationships; the presented package provides a uniform and accessible interface and output for mapping identifiers, facilitating their integration into complex automated workflows. Its technical functionalities such as cache layer, error handling and input vectorisation differentiate it from similar packages and make it more efficient and reliable.

## 2 Data sources

The biological sequence databases integrated by *ginmappeR* are:

1. **CARD** (McArthur *et al.* 2013): the Comprehensive Antibiotic Resistance Database is a database providing up-to-date information on antibiotic resistance mechanisms. It is offered as a centralized repository of curated data on resistance genes, mutations and resistance conditions. CARD aims to facilitate research in tracking, understanding and combating antibiotic resistance.
2. **NCBI** (Sayers *et al.* 2022): the National Center for Biotechnology Information is an aggregation of several resources on genetical and molecular information such as PubMed and GenBank databases, or the BLAST (Altschul *et al.* 1990) sequence alignment tool. The NCBI Protein database offers information on protein sequences, structures, functions and relationships, facilitating research on drug discovery and protein biochemistry. On the other hand, NCBI Nucleotide belongs to the GenBank database and contains nucleotide sequences, both DNA and RNA, from a variety of organisms. The NCBI Gene database stores annotations on genes of different organisms, including symbols, names, location in the genome and associated phenotypes or diseases.
3. **UniProt** (Consortium 2023): the Universal Protein Resource is a protein sequence database maintained by several Bioinformatics organisations that offers sequences, annotations, and protein domains data.
4. **KEGG** (Kanehisa and Goto 2000): the Kyoto Encyclopaedia of Genes and Genomes is a comprehensive database and resource that integrates information on biological pathways, metabolites, diseases, genomes, and organisms. It is a valuable source of complex biological processes at molecular level.

In terms of covered species, NCBI, UniProt, and KEGG contain information on all types of organisms and species, while CARD, focusing on antibiotic resistance data, is limited to bacterial information.

All of the databases provide publicly accessible APIs and have community-developed R packages, except for CARD, which must be used locally. *ginmappeR* handles downloading the compressed CARD file from the official server and decompresses it in R’s temporary folder, ensuring that the latest version is retrieved with each session. The decompression is managed using the untar() function from R’s utils package, which has been tested to work correctly on Windows, MacOS, and Linux. Additionally, *ginmappeR* offers users the option to manually upgrade to the latest version or change the download and decompression path if they have limited access to their machine. When CARD is already available locally, *ginmappeR* loads the aro_index.tsv file into memory and use it to perform translations between CARD and NCBI identifiers.

*ginmappeR* accesses the latest available online version of the databases and downloads the latest update of the CARD database. However, the user can always find out the version used with functions getCARDVersion(), getNCBIVersion(), getUniProtVersion() and getKEGGVersion().

### 2.1 Existing tools

Similar tools can be found in the literature, such as the DAVID Gene ID Conversion Tool (https://davidbioinformatics.nih.gov/conversion.jsp), a web-based utility that offers interoperability between different databases. However, it provides a less user-friendly interface, resulting in a steep learning curve. Additionally, integrating it into a typical R workflow is challenging due to the outdated RDAVIDWebService (Fresno and Fernandez 2013) package. The DAVID conversion tool relies on the DAVID Knowledgebase backend, which aggregates species-specific gene/protein identifiers and their annotations from various public genomic resources like NCBI, UniProt, Ensembl, KEGG, or Reactome, and is updated every three months. The g: Profiler (https://biit.cs.ut.ee/gprofiler/convert) tool and its associated R package (Kolberg *et al.* 2020) offer comparable functionalities but share the same drawback of being updated every few months, while *ginmappeR* performs on-the-fly translations by querying each database, which ensures that users always work with the most current data available.

On the other hand, specific database tools, such as UniProt.ws (Carlson and Ramos 2023), offer utilities for querying UniProt resources and its identifier conversion tool between sequence databases, including NCBI and KEGG. *ginmappeR* utilises this tool, mapUniProt(), to directly convert between UniProt and NCBI identifiers. Another package, UniProtR (Soudy *et al.* 2020), available on CRAN, provides similar functionalities to UniProt.ws but in a more detailed and accessible manner, although the former was chosen because it underwent Bioconductor’s review and acceptance process, which makes it more reliable.

KEGGREST (Tenenbaum 2023) package provides access to the KEGG API and its ID conversion tool, keggConv(), which is more limited but allows *ginmappeR* to perform direct translations between NCBI, KEGG, and UniProt identifiers.

CARD and NCBI databases do not have a dedicated identifier conversion tool. However, rentrez (Winter 2017) NCBI package accesses its web services and make it possible to link between its databases and find equivalences, though this task can be technically challenging and often involves working with XML. *ginmappeR* handles this using rentrez’s entrez_link() and entrez_fetch() functions.

The direct translations obtained using databases tools are accurate but often limited as not all genes or proteins find conversion. Therefore, the significant contribution of *ginmappeR* is working as a central hub that utilises all these tools together, combining them and adding the possibility of using similar or identical genes and proteins when mapping. This significantly increases the success rate of obtaining translations, freeing users from tedious manual tasks while streamlining the workflow and enhancing its flexibility, efficiency, and robustness in gene and protein identifier mapping.

## 3 Methods and implementation

### 3.1 Methods

The R package *ginmappeR* offers several functionalities:

1. Gene and protein identifiers mapping between CARD, UniProt, KEGG and NCBI Protein, Nucleotide and Gene databases. This set of features follows an homogeneous presentation to the user: they are provided as functions with format getX2Y, where X and Y are two of the offered databases, and meaning that a given gene or protein identifier of database X is being translated to database Y. By default, the translation is performed in the most accurate and fastest way, but it is highly configurable since, in some cases, it is possible to obtain multiple translations, including identical proteins or genes, or sequences with a certain percentage of identity.
2. Retrieval of UniProt genes clusters of a certain percentage of similarity, a functionality that is not available in the UniProt.ws R package (Carlson and Ramos 2023) for access to the UniProt database. Function getUniProtSimilarGenes() allows the user to retrieve genes that are 100%, 90% or 50% similar to the one given by its identifier.
3. Retrieval of NCBI identical proteins, a functionality that is not easily accessible and usable in its respective R package rentrez (Winter 2017). Providing a NCBI identifier to the function getNCBIIdenticalProteins(), the user can obtain its identical proteins as a list of identifiers or in a more detailed format such as a dataframe.

### 3.2 Structure

**Figure 1.**
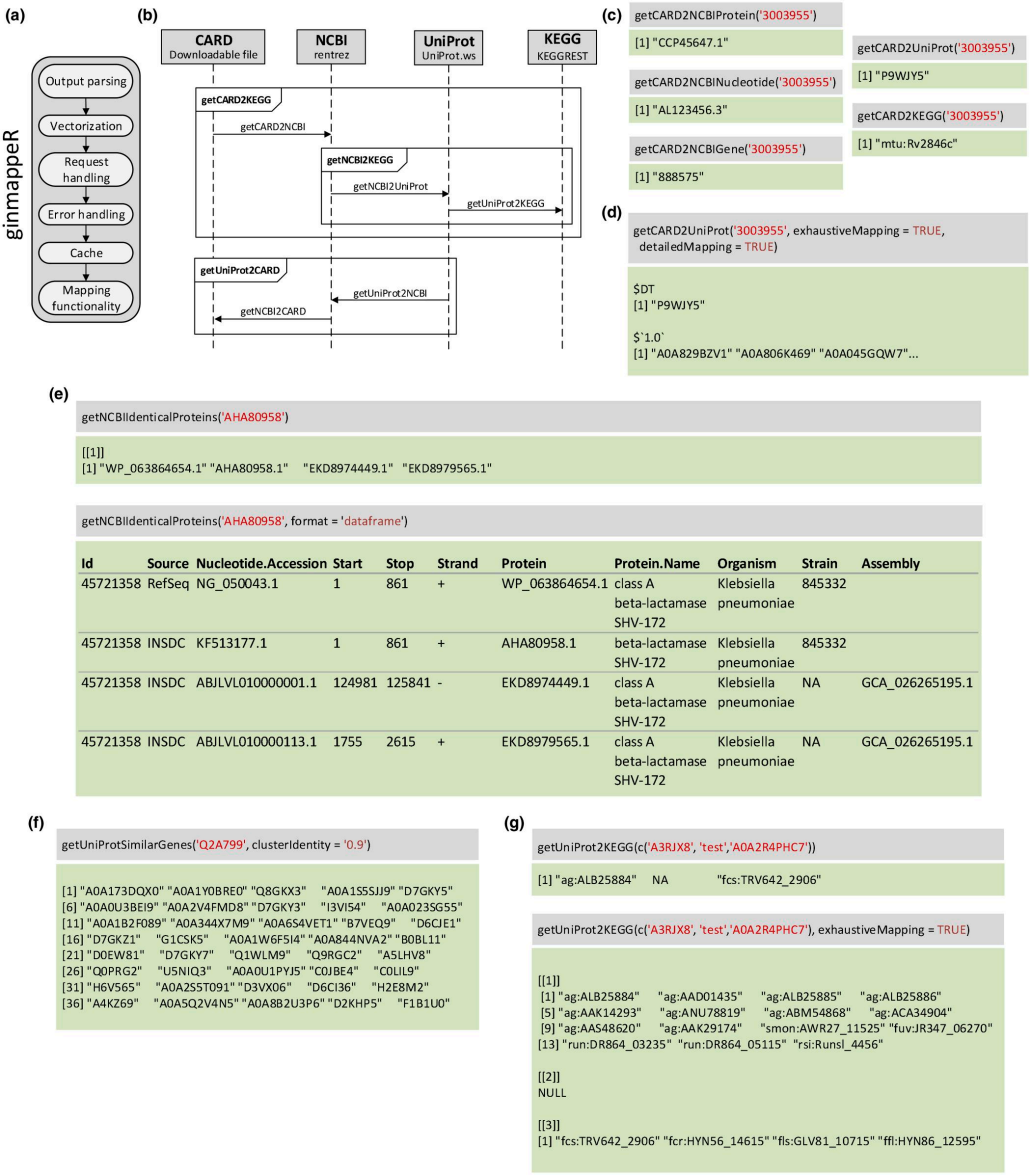
(a) *ginmappeR*’s structure layered design. (b) Example of *ginmappeR*’s functions code reuse. (c) Translations from CARD database to others. (d) Functions’ parameters showcase. (e) Identical proteins retrieval showcase. (f) Similar genes cluster retrieval showcase. (g) Function vectorization and output format showcase.

The implementation of *ginmappeR*’s mapping functions follows a layered design (Fig. 1a), combining code modularity and useful utilities for the user. These layers are:

1. **Mapping functionality**: the core functionality of the package.
2. **Cache layer**: employs the memoise (Wickham *et al.* 2021) and cachem (Chang 2023) R packages to cache both final and intermediate results, reducing response times considerably. This prevents redundant API calls when users request the conversion of sequence IDs previously carried out or a set of genes of the same family, also reducing the risk of API failure.
3. **Error handling**: a layer that manages the different errors produced by the R packages that access the APIs and shows appropriate messages to the user. It also avoids caching incorrect results when APIs fail or are not available.
4. **Request handling**: because of the unstable nature of accessed APIs, this layer handles the requests and is responsible for retrying them several times if an error is raised. This mechanism has little impact on execution time and highly improves the usability of the package.
5. **Vectorization**: this layer allows the user to map a vector of several identifiers in a single call. This is achieved by using the R base Vectorize() function.
6. **Output parsing**: takes care of formatting returned results in a homogenized way following R functions conventions.

### 3.3 Implementation

Reuse of code plays a main role in *ginmappeR* as the translation from a database to another one usually implies using an intermediate one. Consequently, functions provided by *ginmappeR* often make use of other functions to obtain mid-process translations. Figure 1b shows how function getCARD2KEGG() first uses getCARD2NCBI(), and after that, translates the NCBI identifier to KEGG through getNCBI2KEGG(). This principle applies in both directions of each mapping process: For example, when translating from UniProt to CARD, the getUniProt2CARD() function’s workflow is composed by getUniProt2NCBI() and getNCBI2CARD().

In terms of input and output formats, *ginmappeR* follows the standard behaviour and widely consensual approach of vectorized R functions, such as sqrt() or toupper(), allowing the user to provide a single identifier or a vector character containing several of them. The output format depends on the function and parameters chosen, but also follows the R convention, returning NA values when no translation is found or an error occurs. When the output is a list (triggered by exhaustiveMapping parameter), NA values are replaced by NULL to ensure consistency in the use of functions such as lenghts() to process the result by the user.

## 4 Usage example

To showcase *ginmappeR*’s functionalities, the CARD identifier 3003955 is considered, which corresponds to gene *efpA*. Through the use of the functions getCARD2NCBIProtein(), getCARD2NCBINucleotide() and getCARD2NCBIGene() it can be translated to NCBI databases (Fig. 1c). In the same way, functions getCARD2KEGG() and getCARD2UniProt() perform the same operation but for KEGG and UniProt databases, respectively.

Some of the translation functions have parameters to obtain all possible translations (exhaustiveMapping) or to detail the percentage of identity of the source id with the obtained id (detailedMapping). They can be used when translating to UniProt (Fig. 1d). Further explanation on these and other parameters are to be found on package’s documentation.

The retrieval of identical proteins in the NCBI database offers the result as a list of identifiers or as a more detailed dataframe format, depending on parameter format (Fig. 1e). Obtaining the cluster of genes of a certain percentage of similarity (Fig. 1f) results in a vector with their identifiers.

When providing a vectorised input of several identifiers, a character vector of the same length with the translations will be returned. If parameter exhaustiveMapping is TRUE, it will return a list instead of a character vector to avoid mixing the identifiers, as it may retrieve more than one for each input gene or protein (Fig. 1g).

## 5 Conclusions

In this application note, we have introduced *ginmappeR*, a comprehensive R package that provides a unified and accessible interface for gene and protein identifier mapping. By integrating various biological sequence databases, *ginmappeR* addresses the challenges of database heterogeneity and low coupling, enabling users to seamlessly work with multiple data sources in a cohesive and efficient manner. Additionally, *ginmappeR* offers unique utilities, such as accessing UniProt similar genes and NCBI identical proteins, which enhance the flexibility and richness of the mapping process. Furthermore, its advanced features, such as caching for both final and intermediate translations and robust error handling, set it apart from other packages and help reducing processing times and minimising network traffic, especially when translating large volumes of genes or proteins.

## Data Availability

The data underlying this article are available in NCBI, UniProt, KEGG and CARD databases at https://www.ncbi.nlm.nih.gov/, https://www.uniprot.org/, https://www.kegg.jp/ and https://card.mcmaster.ca/.
